# Gender Differences and Risk Factors of Recurrent Stroke in Type 2 Diabetic Malaysian Population with History of Stroke: The Observation from Malaysian National Neurology Registry

**DOI:** 10.1155/2019/1794267

**Published:** 2019-12-11

**Authors:** Sohail Aziz, Siti Maisharah Sheikh Ghadzi, Nur Ezzati Abidin, Balamurugan Tangiisuran, Hadzliana Zainal, Irene Looi, Khairul Azmi Ibrahim, Norsima Nazifah Sidek, Loo Keat Wei, Lee Keng Yee, Zariah Abdul Aziz, Sabariah Noor Harun

**Affiliations:** ^1^School of Pharmaceutical Sciences, Universiti Sains Malaysia, 11800 USM, Penang, Malaysia; ^2^Pusat Racun Negara, Universiti Sains Malaysia, 11800 USM, Penang, Malaysia; ^3^Clinical Research Centre, Seberang Jaya Hospital, Ministry of Health, Penang, Malaysia; ^4^Clinical Research Centre, Hospital Sultanah Nur Zahirah, Ministry of Health, Terengganu, Malaysia; ^5^Department of Biological Science, Faculty of Science, Universiti Tunku Abdul Rahman, Bandar Barat, 31900 Kampar, Perak, Malaysia; ^6^National Institutes of Health (NIH), Ministry of Health, Malaysia, Kuala Lumpur, Malaysia

## Abstract

**Background and Purpose:**

Diabetes mellitus has been reported as a strong independent risk factor for stroke recurrence. Data on the modifiable factors contributing to the recurrence of stroke in type 2 diabetic Malaysian population with a history of stroke stratified by genders are lacking, and this supports the importance of this study.

**Method:**

The data of 4622 patients with T2DM who had a history of stroke was obtained from the Malaysian National Stroke Registry. Univariate analysis was performed to differentiate between genders with and without stroke recurrence in terms of demographics, first stroke attack presentations, and other clinical characteristics. The significant factors determined from the univariate analysis were further investigated using logistic regression.

**Results:**

Ischemic heart diseases were found significantly associated with the stroke recurrence in males (OR = 1.738; 95% CI: 1.071-2.818) as well as female (OR = 5.859; 95% CI: 2.469-13.752) diabetic patients. The duration of hypertension, as well as the duration of diabetes, has been associated with the recurrence in both male and female subjects (*p* value < 0.05). Smoking status has an impact on the stroke recurrence in male subjects, while no significant association was observed among their peers.

**Conclusions:**

Most of the predictive factors contributing to the recurrence of stroke in type 2 diabetic Malaysian population with a history of stroke are modifiable, in which IHD was the most prominent risk factor in both genders. The impact of optimizing the management of IHD as well as blood glucose control on stroke recurrence may need to be elucidated. No major differences in recurrent stroke predictors were seen between genders among the Malaysian population with type 2 diabetes mellitus who had a previous history of stroke.

## 1. Introduction

According to the 2016 survey, 5.53 million people worldwide suffered from stroke. On the basis of premature mortality and secondary disabilities, stroke was the second highest in ranking [1]. Based on epidemiological data, patients with diabetes mellitus (DM) are at a higher risk of developing atherosclerotic vascular disorders than individuals without DM [2]. Prospective studies have shown that hyperglycemia plays a vital role in the development of vascular complications [3]. Although the impact of diabetes and hyperglycemia on stroke can be observed in various studies [3, 4, 7], there is a lack of defining factors responsible for strokes in diabetes.

Diabetes is considered one of the most consistent predictors of recurrent stroke. Diabetic patients carry an astounding three-fold risk for recurrent stroke in comparison to normoglycemic individuals [4]. Patients with diabetes have a cluster of established risks, such as hypertension, dyslipidemia, and obesity that contribute to stroke [5]. An estimated one-third of acute stroke patients have been found to be hyperglycemic at the time of presentation, with increased mortality and morbidity in the immediate poststroke period [6]. As observed in earlier studies, the effects of DM were worst in females than in males [7]. These deteriorating effects are associated with endothelial dysfunction and more severe hypertension in diabetic females as well as the inflammatory effects of diabetes, which play an important role in stroke prevalence and in aggravating the damage to the brain from stroke [8, 9]. Understanding these gender-based factors is indeed important in minimizing the risk factors associated with diabetes as well as poststroke complications.

An intriguing relationship can be seen between diabetes, stroke, and gender of the individuals. Based on data from a meta-analysis, it has been estimated that diabetic female carries a 27% higher risk of stroke in comparison to diabetic male [9]. Some other studies suggest that the survival and functional outcomes in females are worse than those in males [10–12]. Similarly, hypercholesterolemia, smoking, physical inactivity, obesity, hypertension, and a family history of stroke in a patient with diabetes can be considered major risk factors for the development of recurrent stroke [13]. Although all of these factors can individually contribute to the recurrence of stroke, a systematic analysis reported that 90% of stroke burden from 1990 to 2013 was caused by the combined effects of all these modifiable factors [12]. Despite the available research based on the impact of diabetes on stroke recurrence, the defining factors which contribute to the stroke recurrence in diabetes patients are still lacking. Based on gender, our study focused on multiple factors that contribute to stroke recurrence in the Malaysian population with type 2 diabetes mellitus (T2DM).

## 2. Aim of the Study

The aims of the current study were (a) to determine the prevalence of recurrent stroke in type 2 diabetic Malaysian population with a history of stroke and (b) to differentiate all those intriguing factors which contribute to the recurrence of stroke in T2DM patients based on gender.

## 3. Ethics Approval

The ethical approval for this study was obtained from the Medical Research and Ethics Committee (MREC), Ministry of Health, Malaysia.

## 4. Method

### 4.1. Patients' Data Collection

All patients' data from August 2009 to December 2016 were extracted from the National Stroke Registry of Malaysia. Data of patients who were diagnosed with type 2 diabetes mellitus (T2DM) and had a history of stroke were extracted from the registry and included in the analysis. The clinical diagnosis of stroke was made according to the World Health Organization's criteria [7]. All diagnoses were confirmed using brain computed tomography or magnetic resonance imaging. The patient's neurological function deficit was evaluated using the National Institute of Health Stroke Scale (NIHSS), Barthel index (BI), and the modified rank scale (mRS) on admission. Stroke severity was categorized using the NIHSS into three groups: mild (NIHSS: ≤7), moderate (NIHSS: 8–16), and severe (NIHSS: ≥17) [14]. Diabetes was either defined as self-reported physician diagnosis or based on the use of hypoglycemic medications prior to the first stroke attack during hospitalization secondary to stroke attack or at discharge. Similarly, hyperlipidemia and ischemic heart disease were also defined either by self-report or by the medications prescribed. Patients were followed up for a minimum of 1 year for clinical outcomes of recurrent stroke, death, and functional outcome.

The recurrent stroke risk factors included the medical history of hypertension, diabetes mellitus (DM), dyslipidemia, atrial fibrillation (AF), and ischemic heart disease (IHD). We also evaluated modifiable lifestyle factors, including current smoking (≥1 cigarette per day for ≥1 year), alcohol consumption (≥1 drink per week for 1 year), and obesity [body mass index (BMI) ≥ 30 kg/m^2^].

### 4.2. Stroke Registry in Malaysia

The multiethnic National Neurology Registry (NNEUR) in Malaysia was first established in 2009. The NNEUR has collected information on stroke cases from 13 states in the country. NNEUR represents an ongoing multicenter, hospital-based registry that is aimed at providing comprehensive epidemiological data on the country's stroke statistics, trend, and management. The registry is funded by the Ministry of Health, Malaysia (MOH), with support from the National Network Clinical Research Centre, MOH. The complete details on the stroke registry (http://acrm.org.my/nneur/) have been previously described elsewhere [27].

### 4.3. Analysis

The cross-sectional analysis employed univariate and multivariate logistic regression to evaluate the association between explanatory factors and recurrent stroke using SPSS version 22.0 (SPSS Inc., Chicago, III, USA). The values for continuous variables were expressed as the mean ± standard deviation, while categorical variables were presented in percentage. A chi-square test was used for the univariate analysis of categorical variables while an independent sample *t*-test/Mann–Whitney *U* test was used for continuous data. The significant variables from the univariate analysis were further analyzed using multiple logistic regression. The association between exposure and outcomes was reported as an odds ratio (OR) with a 95% confidence interval (CI). In order to minimize bias from missing data, the pattern of missing values of independent variables was analyzed. Multiple imputations were used to handle variables with missing values above 5%. Missing values in BMI, prior medications, uric acid level on the first stroke event, and total cholesterol level on the first stroke event were imputed from multivariate imputation. Five imputations were used, and Rubin's rules were implemented to combine the results. A two-tailed probability value of *p* < 0.05 was considered significant.

## 5. Results

### 5.1. Descriptive Results

Overall, 4622 diabetes patients who had a history of stroke were included in the analysis. As shown in Table 1, from the total diabetic patients with a history of stroke, 2280 (49.32%) were female, age < 65 years (54.3%). The ischemic stroke was the most prominent type of stroke in both genders. The majority of patients in both genders were diagnosed with diabetes for 1-5 years. 1513 male patients (64.6%) were smokers, and those with ischemic heart diseases (16.9%) were the most with prevalent concomitant disease in this population as compared to their counterparts. 2063 female patients (90.5%) had hypertension while 753 (33.0%) had hyperlipidemia. The mortality was found to be less prevalent in females (9.1%) as compared to male patients (12.2%), as recorded in Table 1.

### 5.2. Factors Associated with Stroke Recurrence in Female Diabetic Patients with History of Stroke

Associations between the variables and stroke recurrence in diabetic female patients with a history of stroke are represented in univariate and multivariate logistic regression as shown in Table 2. An independent association was found between IHD and stroke recurrence among diabetic female patients with a history of stroke in the final logistic regression model. A history of IHD prior to the first-time stroke attack increased the odds of having recurrence of stroke (OR, 5.859; 95% CI [2.49-13.75]; *p* < 0.001). Discharge medications with diuretic therapy from admission during the first-time stroke were associated with increased odds of stroke recurrence (OR, 3.476; 95% CI [1.146-10.538]; *p* < 0.05). The presence of seizure at the first-time stroke attack predicted the increased risk of stroke recurrence (OR, 4.641; 95% CI [1.126-19.126]; *p* < 0.05), and an increase in every 1 mmHg of blood pressure was associated with an increased odd of having recurrent stroke (OR, 1.017; 95% CI [1.006-1.028]; *p* < 0.05). It was found that among diabetic females, duration of diabetes and hypertension, and patients with hyperlipidemia were statistically significant in the univariate analysis. However, they were not significant after adjusting with other covariates using multivariate analysis. Similarly, hyperlipidemia was significant in the univariate analysis; however, the significance level was removed from the final regression model after considering other variables (Table 2).

### 5.3. Factors Associated with Stroke Recurrence in Diabetic Male Patients with History of Stroke

Associations between variables and stroke recurrence in diabetic male patients with a history of stroke are presented in Table 3. As in female patients, being diagnosed with IHD prior to the first-time stroke attack was also a significant prediction of recurrent stroke among males with diabetes (OR, 1.738; 95% CI [1.017-2.818]; *p* < 0.001). In the ethnicity group in the subcategory “others” originating from East Malaysia, the odds of stroke recurrence was significantly less among males with diabetes (OR, 0.330; 95% CI [0.207-0.528]; *p* < 0.001). Male patients who have been diagnosed with diabetes for a duration of less than a year and more than a decade were found to have a significant increase in the odds of stroke recurrence in the final regression model (OR, 2.917; 95% CI [1.007-8.454]; *p* < 0.05) and (OR, 2.917; 95% CI [1.163-11.466]; *p* < 0.05), respectively (Table 3). In addition, having had a clinical manifestation of monoparesis at the first-time stroke event increased the probability of stroke recurrence (OR, 4.076; 95% CI [1.230-13.506]; *p* < 0.05). Nevertheless, experiencing an altered sensorium during the first-time stroke event reduced the probability of stroke recurrence (OR, 0.172; 95% CI [0.41-0.724]; *p* < 0.05) among diabetic male patients with a history of stroke.

## 6. Discussion

This current study is based on a multiethnic Malaysian population, which to our knowledge, is the first study focusing on the associated predictors of recurrent stroke in the type 2 diabetic Malaysian population with a history of stroke stratified by gender. According to a study conducted in Japan on the recurrence of stroke, it has been observed that some predictors of stroke are time-specific and may differ in late and early recurrences, such as T2DM which is considered a specific predictor of late recurrence [15]. Most studies based on the predictors of recurrent stroke were conducted in the general population, and diabetes was commonly found as the significant risk factor [4]. Moreover, those studies were mostly done in the white population, with the data being extrapolated to other population groups [6–8]. As in other Asian populations, ischemic stroke was identified as the most common type among the Malaysian diabetic populations [16]. However, there was no difference found in the incidence of recurrent stroke between males (4.6%) and females (4.8%) in our population, which contradicted the findings from previous studies [17, 18]. This suggests that the overall difference between genders for the risk of recurrent stroke stratified by diabetes is still inconclusive. A possible explanation of this might be that the distribution of age range in both genders is similar, which was <65 years old. As shown previously, a diabetic male aged <75 years has a higher risk of recurrent stroke than a female [7].

As shown in the current study, IHD was the independent predictor of recurrent stroke in both genders. This observation was in concordance with previous studies, suggesting that factors such as malfunctioning nitric oxide (NO) system, hypercoagulability, and endothelial dysfunction in patients with diabetes having IHD are responsible for the recurrence of stroke [19–22]. The NO system protects vessels from platelet aggregation. An increased metabolism of NO or a decrease in production can lead to hypercoagulability and narrowing of vessels, which thus contributes to ischemia and any increase in the metabolism of NO, or its decrease production leads to hypercoagulability and narrowing of vessels [19]. Furthermore, impairment in fibrinolysis markers, coagulation factors, and inflammatory markers in diabetes patients also contributes directly to the recurrence of stroke [22]. This is also suggesting that irrespective of gender, IHD is the modifiable indicator for the high risk of the occurrence of recurrent stroke. Further studies to determine the extent of optimal therapy for IHD that may reduce the risk of recurrent stroke may be warranted.

Surprisingly, in contrast to earlier findings in other Asian populations [23, 24], no significant association between hyperlipidemia and recurrent stroke was found in our diabetic population irrespective of gender groups. Nevertheless, our findings are consistent with those of Greater Cincinnati/Northern Kentucky Stroke Study (GCNKSS) and Atherosclerosis Risk in Communities (ARIC) study which did not support hyperlipidemia as a predictor of recurrent stroke in diabetic population [25, 26].

A wide range of available published data links cardiovascular system (CVS) disorders with T2DM in comparison to type 1 diabetes which accounts for only 10-20% of the overall population [13, 15, 16]. It has been observed in previous works that female patients have a lower risk to develop stroke than male patients even in the presence of risk factors like hypertension and diabetes [27, 28]. This can be associated with the neuroprotective effects of estrogen, progesterone, and sex steroidal hormones in females [29, 30]. However, there are also some contradictory studies based on gender reported that female patients with diabetes are at a comparatively higher risk of developing ischemic stroke in comparison to male subjects [9–11]. Despite all of the previous findings, our study did not observe any association between CHD and gender with the occurrence of recurrent stroke.

Hypertension is one of the classical risk factors for the development of stroke in diabetic patients. Without considering other variables into account, the extended duration of hypertension was found to be significantly associated with recurrent stroke in both male and female diabetic patients in the current study. Nevertheless, every 1 mmHg increase in blood pressure increases the risk of recurrent stroke in female diabetic patients. This result is consistent with a previous study that reported that female patients are comparatively more prone to hypertension and are more prone to recurrent stroke [23]. Intensive blood pressure control has been shown to be associated with a significant reduction in total stroke in the general population [31, 32]. Diabetic patients benefited from the antihypertensive treatment as observed in the Systolic Hypertension in Europe Trial with a 73% decrease in stroke incidence in diabetes patients as compared to a 38% decrease in nondiabetic patients by targeting the systolic blood pressure [33]. However, whether the female diabetic population requires more aggressive blood pressure control as compared to males to reduce recurrent stroke may need to be further investigated.

In accordance with the current study, it has been observed that the duration of diabetes significantly increases the chance of stroke [34]. The effects of diabetes duration on the risk of recurrent stroke have been observed in univariate analysis; however, no significant effects were observed by using multivariable analysis. A prospective cohort study was performed to determine the role of duration of diabetes and its impact on stroke, and the authors have reported that the risk of stroke increased by 3% every year and tripled after ten years of diabetes duration [35]. The maximum observed risk was after ten years of diabetes which supports the results of the current study. Many potential mechanisms like atherosclerotic lesions, the prevalence of hypertension, increased microalbuminuria, and endothelial dysfunction, which is directly related to the duration of diabetes, can be associated with the increased risk of stroke [36–38]. Furthermore, our study has observed an increase in odds of stroke recurrence for patients with less than one year of diabetes duration. In accordance to the current study results, a study performed on Canadian population has observed that newly treated diabetes patients in their first five years have a 10% absolute risk of recurrent stroke in comparison to general population [47]. However, the current study lacks the details about the age or concomitant disease conditions of those newly diagnosed diabetes patients which may be associated with the increase odds of recurrence.

Similarly, on the basis of smoking status, a significant association has been found in the current study which relates smoking status to the increased chances of recurrent stroke. The result was significant in the univariate analysis but was not in the multivariable logistic regression. It has been observed that current smokers are at a higher risk of recurrence stroke, in which males are predominant to recurrence if they are active smokers, as compared to females. As observed in a previous study, smoking increases the risk associated with diabetes and has an impact on the lipid profile of the smoker, hence increasing the risk of stroke recurrence [39]. In accordance with the current study, a study based on the effects of multiple factors on recurrence stroke has observed an association between smoking in males with the recurrence of stroke or death associated with stroke [40]. Still, there is a lack of established literature based on the effect of smoking on stroke recurrence in diabetic patients, and this might be associated with the low number of diabetic patients who smoke.

In the current study, it has been observed that treatment strategies targeting hypertension and hyperglycemia are significantly associated with the prevention of the incidence of recurrent stroke. In the present study, receiving diuretics upon discharge from hospitalization secondary to the first stroke attack was found to increase the odds of recurrent stroke among diabetic females. In common practice, diuretics were commonly indicated for fluid overload management either secondary to heart failure or renal dysfunction which indirectly influences the blood pressure control. Based on a study finding, the variability in the blood pressure may contribute to the recurrence of stroke and low SBP increased the risk of poor outcomes [45]. All these factors can be associated with the use of diuretics which has shown a higher degree of decrease in the blood pressure [46] and may be the reason behind the poor outcomes in the present study which need further elucidated approaches for better understanding of the scenario.

Oral hypoglycemic agents in both male and female patients show a significant beneficial effect on the lower risk of recurrence stroke. Based on the literature supporting the results of the current study, it has been summarized that stroke recurrence can be prevented by the management of risk factors, e.g., hypertension through optimizing the treatment strategies [41]. As diabetes carries the risk of microvascular as well as macrovascular complications, causing an increase in cardiovascular disorders and hence antihypertensive therapy either alone or in combination plays a vital role in reducing the absolute risk of stroke [42]. In contrast to the results of the current study where beta-blockers and diuretics have been found effective, the Intervention as a Goal in Hypertension Treatment (INSIGHT) study suggests that older antihypertensive agents are more likely to impair glucose metabolism in comparison to newer antihypertensive like ACEI which can directly influence the outcomes in diabetic patients [43]. Tight control on hyperglycemia could effectively improve microvascular complications, but its impact on macrovascular complications as well as stroke is still lacking. A former study supports the results of the current study, which suggests that effective glycemic control with oral hypoglycemic agents like metformin can effectively help in the prevention of stroke in diabetes patients [44]. UKPDS trials suggest that glycemic control with hypoglycemic agents in the intensive group has no significant benefits in the reduction of stroke incidence (*p* = 0.52) [45].

## 7. Conclusions

Most of the predictive factors contributing to the recurrence of stroke in diabetic Malaysian populations with a history of stroke are modifiable in which IHD was the most prominent risk factor in both genders. The impact of optimizing the management of IHD as well as blood glucose control on stroke recurrence may need to be elucidated. No major differences in recurrent stroke predictors were seen between genders among type 2 diabetic Malaysian populations with a history of stroke.

## 8. Limitations

The current study was retrospective based on the data available from the National Neurology Registry of Malaysia. On the basis of ethnicity, a large number of contributing data were patients from the “others” category which includes the people from Eastern Malaysia. Moreover, diabetic patients with a history of ischemic stroke were more prevalent in the study than other types of stroke and hence there can be bias in generalizing the data to overall stroke incidences in diabetes patients. In our study, the data related to medications at discharge which were taken continuously or were newly prescribed was unknown. Furthermore, the data related to the blood glucose control (e.g. glycated hemoglobin (HbA1c)) was not available which limits our ability to determine the association between blood glucose control and stroke recurrence. Therefore, based on the limitations, we were not able to establish whether the risks were associated with differences in care, worse glycemic control, or poor management after the occurrence of the first stroke event as suggested by others.

## Figures and Tables

**Table 1:** Characteristic of female and male diabetic patients presented as the number of subjects in each category (*N* = 4622).

Variables	Female (*n* = 2280)		Male (*n* = 2342)	
	*N*	%	*N*	%
WHO classification				
Intracerebral hemorrhage	265	11.6	300	12.8
Ischemic stroke	1924	84.4	1962	83.8
Subarachnoid hemorrhage	7	0.3	5	0.2
Transient ischemic attack	63	2.8	56	2.4
Unclassified	20	0.9	19	0.8
Age (years)				
<65	1237	54.3	1420	60.6
≥65	1043	45.7	919	39.3
Ethnicity				
Malay	558	24.5	533	22.8
Chinese	69	3.0	74	3.2
Indian	48	2.1	36	1.5
Others	1605	70.4	1699	72.5
Education				
Informal	988	43.3	795	33.9
Formal	1292	56.7	1547	66.1
Marital status				
Single	36	1.6	52	2.2
Married	1982	86.9	2061	88.0
Divorced	14	0.6	17	0.7
Widowed	96	4.2	18	0.8
Unknown	152	6.7	194	8.3
Smoker	856	37.5	1513	64.6
Duration of diabetes (years)				
>1	155	6.8	172	7.3
1-5	861	37.8	885	37.8
6-10	365	16.0	323	13.8
>10	377	16.5	319	13.6
Unknown	522	22.9	643	27.5
BMI				
Normal	423	18.6	365	15.6
Overweight	601	26.4	599	25.6
Obese	1192	52.3	1293	55.2
Underweight	52	2.3	58	2.5
Hypertension	2063	90.5	1993	85.1
IHD	235	10.3	395	16.9
Hyperlipidemia	753	33.0	637	27.2
Atrial fibrillation	68	3.0	72	3.1
Death	208	9.1	285	12.2

Abbreviations: BMI = body mass index; IHD = ischemic heart disease; WHO = World Health Organization.

**Table 2:** Univariate and multivariate logistic regression analyses of variables associated with stroke recurrence in female patients with diabetes.

	Univariate analysis			Logistic regression^∗^	
	Without recurrence *N* = 2171 *N* (%)	With recurrence *N* = 109 *N* (%)	*p* value	OR [95% CI]	*p* value

Constant	—	—	*—*	0.217	*0.763*

Age (years)					
<65	1165 (53.6)	72 (66.1)			
≥65	1006 (46.4)	37 (33.9)	0.013	—	*—*
Duration of diabetes					
<1 year	147 (6.8)	8 (7.3)	0.005	Reference	
1-5 years	810 (37.3)	51 (46.8)		1.537 [0.229-10.318]	0.658
6-10 years	347 (16.0)	18 (16.5)		1.161 [0.153-8.823]	0.885
>10 years	354 (16.3)	23 (21.1)		1.334 [0.165-10.780]	0.787
Unknown	513 (23.6)	9 (8.3)		0.291 [0.018-4.793]	0.388
Education					
Formal	1204 (55.4)	88 (80.7)	0.000	—	—
Informal	967 (44.6)	21 (19.3)			
Mode of arrival at emergency at the first-time stroke attack^∗^				—	—
Ambulance	1036 (47.7)	71 (65.1)	0.001		
Own transport	1026 (47.2)	37 (34.0)			
Unknown	109 (5.1)	1 (0.9)			
Duration of hypertension				Reference (0 years)	
<1 year	105 (4.8)	5 (4.5)	0.006	1.488 [0.147-15.040]	0.736
1-5 years	720 (33.1)	45 (41.5)		2.078 [0.184-23.417]	0.554
6-10 years	326 (15.0)	18 (16.5)		1.875 [0.171-20.539]	0.607
>10 years	372 (17.1)	27 (24.7)		2.137 [0.105-43.543]	0.622
Unknown	437 (19.3)	8 (7.3)		1.374 [0.117-16.184]	0.801
No hypertension	211 (9.7)	6 (5.5)			
Smoking status					
Current smoker	28 (1.3)	0 (0.0)	0.401	—	—
Former smoker	27 (1.2)	1 (0.9)			
Never	2089 (96.3)	108 (99.1)			
Unknown	27 (1.2)	0 (0.0)			
Hyperlipidemia	707 (93.9)	46 (6.1)	0.025	—	—
Ischemic heart disease	212 (90.2)	23 (9.8)	0.001	5.859 [2.496-13.752]	0.000
*ACEIs* received at baseline^∗∗^	2088 (95.4)	101 (4.6)	0.066	—	—
*ARBs* received at baseline^∗∗^	2105 (95.4)	101 (4.6)	0.023	—	—
ADM received at baseline^∗∗^	909 (93.1)	67 (6.9)	0.000	1.409 [0.467-4.251]	0.542
Received OHA upon discharge^∗∗∗^	726 (93.3)	52 (6.7)	0.002	0.909 [0.327-2.525]	0.855
Received diuretics upon discharge^∗∗∗^	143 (89.9)	16 (10.1)	0.003	3.476 [1.146-10.538]	0.028
Glasgow Coma Scale: motor response	58 (2.7)	1 (0.9)		—	—
No response			0.006		
Extension response in response to pain	15 (0.7)	4 (3.7)			
Flexion in response to pain	31 (1.4)	0 (0.0)			
Withdraws in response to pain	46 (2.1)	1 (0.9)			
Purposeful movement to painful stimulus	244 (11.4)	8 (7.4)			
Obeys commands for movement	1747 (81.6)	94 (87.0)			
Apparent clinical manifestations at baseline^∗∗^					
Altered sensorium	220 (97.8)	5 (2.2)	0.028	—	—
Monoparesis	32 (84.2)	6 (15.8)	0.010	—	—
Seizure	40 (87.0)	6 (13.0)	0.016	4.641 [1.126-19.126]	0.034
Systolic blood pressure (mmHg)	2171	109	0.000	1.017 [1.006-1.028]	0.002
(Mean ± SD)	(167.96 ± 34.07)	(180.65 ± 34.35)			
Total cholesterol l (mmol/L)	2171	109	0.008	1.103 [0.942-1.291]	0.222
(Mean ± SD)	(6.03 ± 1.82)	(6.52 ± 2.45)			
NIH stroke scale at baseline^∗∗^: consciousness	1265 (81.5)	74 (92.5)		—	—
Alert			0.067		
Sleepiness	174 (11.2)	5 (6.3)			
Stupor	72 (4.6)	0 (0.0)			
Coma	42 (2.7)	1 (1.3)			
NIH stroke scale at baseline^∗∗^: visual field					
No visual loss	1207 (79.5)	61 (77.2)	0.033		
Partial hemianopia	175 (11.5)	16 (20.3)		—	—
Complete hemianopia	94 (6.2)	2 (2.5)			
Bilateral hemianopia	43 (2.8)	0 (0.0)			

^∗^Significant variables remained in the final logistic regression model,^∗∗^ received prior to the first-time stroke attacks^∗∗∗^ discharged from admission during the first-time stroke event; ^∗∗∗∗^mean ± standard deviation. Abbreviations: ACEIs = angiotensin-converting enzyme inhibitors; ARBs = angiotensin receptor blockers; ADM = antidiabetic medications; NIH=National Institute of Health; OHA = oral hypoglycemic agent; SD = standard deviation.

**Table 3:** Univariate and multivariable logistic regression analysis of variables associated with stroke recurrence in male patients with diabetes.

	Univariate analysis			Logistic regression^∗^	
	Without recurrence *N* = 2234 *N* (%)	With recurrence *N* = 108 *N* (%)	*p* value	OR [95% CI]	*p* value

Constant	—	—	*—*	0.108	0.001

AGE (years)					
<65	1345 (60.3)	75 (69.4)	*0.069*	—	—
≥65	886 (29.7)	33 (30.6)			
Duration of diabetes					
<1 year	167 (7.5)	5 (4.6)	0.009	Reference	
1-5 years	831 (37.2)	54 (50.0)		2.917 [1.007-8.454]	0.049
6-10 years	309 (13.8)	14 (13.0)		2.540 [0.782-8.244]	0.121
>10 years	294 (13.2)	25 (23.1)		3.652 [1.163-11.466]	0.027
Unknown	633 (28.3)	10 (9.3)		1.277 [0.343-4.752]	0.715
Education					
Formal	1461 (65.4)	86 (79.6)	0.001	—	—
Informal	773 (34.6)	22 (20.4)			
Ethnicity					
Malay	479 (21.4)	54 (50.0)		Reference	
Chinese	70 (3.1)	4 (3.7)	0.000	0.714 [0.231-2.208]	0.559
Indian	33 (1.5)	3 (2.8)		0.827 [0.212-3.228]	0.784
Others^#^	1652 (73.9)	47 (43.5)		0.330 [0.207-0.528]	0.0001
Mode of arrival at emergency upon the first-time stroke attack^∗^					
Ambulance	992 (44.4)	43 (39.8)	0.026	—	—
Own transport	1104 (49.4)	64 (59.3)			
Unknown	138 (6.2)	1 (0.9)			
Duration of hypertension				Reference (0 years)	
<1 year	116 (5.2)	7 (6.5)	0.005	1.488 [0.147-15.040]	0.736
1-5 years	721 (32.3)	40 (37.0)		2.078 [0.184-23.417]	0.554
6-10 years	279 (12.5)	11 (10.2)		1.875 [0.171-20.539]	0.607
>10 years	268 (12.0)	20 (18.5)		2.137 [0.105-43.543]	0.622
Unknown	522 (23.4)	9 (8.3)		1.374 [0.117-16.184]	0.801
Smoking status					
Current smoker	518 (23.2)	40 (37.0)	0.002	—	0.85
Former smoker	432 (19.3)	19 (17.6)		1.452 [0.907-2.325]	0.120
Never	1171 (52.4)	49 (45.4)		0.678 [0.379-1.213]	0.190
Unknown	113 (5.1)	0 (0.0)		—	0.99
Hyperlipidemia	602 (94.5)	35 (5.5)	0.224	—	—
Ischemic heart disease	368 (93.2)	27 (6.8)	0.025	1.738 [1.071-2.818]	0.025
*ACEIs* received at baseline^∗∗^	2143 (95.9)	91 (4.1)	0.000	0.374 [0.131-1.067]	0.066
*ARBs* received at baseline^∗∗^	2152 (95.9)	93 (4.1)	0.000	1.226 [0.409-3.673]	0.715
ADM received at baseline^∗∗^	913 (93.9)	59 (6.1)	0.005	1.409 [0.467-4.251]	0.542
Received OHA upon discharged^∗∗∗^	748 (94.2)	46 (5.8)	0.034	0.909 [0.327-2.525]	0.855
Glasgow Coma Scale: motor response					
No response	61 (2.8)	1 (0.9)	0.037	—	—
Extension response in response to pain	20 (0.9)	0 (0.0)			
Flexion in response to pain	42 (1.9)	0 (0.0)			
Withdraws in response to pain	37 (1.7)	1 (0.9)			
Purposeful movement to painful stimulus	218 (9.9)	10 (9.3)			
Obeys commands for movement	1820 (82.8)	95 (88.9)			
Apparent clinical manifestations at baseline^∗∗^					
Altered sensorium	242 (99.2)	2 (0.8)	0.001	0.172 [0.41-0.724]	0.016
Monoparesis	25 (86.2)	2 (13.8)	0.046	4.076 [1.230-13.506]	0.022
NIH stroke scale at baseline^∗∗^: consciousness				—	—
Alert	1335 (82.8)	69 (93.2)	0.007		
Sleepiness	161 (10.0)	5 (6.8)			
Stupor	79 (4.9)	0 (0.0)			
Coma	38 (2.4)	0 (0.0)			
NIH stroke scale at baseline^∗∗^: visual field					
No visual loss	1265 (80.8)	62 (83.8)	0.090		
Partial hemianopia	195 (12.5)	5 (6.8)		—	—
Complete hemianopia	77 (4.9)	3 (4.1)			
Bilateral hemianopia	29 (1.9)	4 (5.4)			

^∗^Significant variables remained in the final logistic regression model (highlighted in grey), ^∗∗^ received prior to the first-time stroke attacks ^∗∗∗^ discharged from admission during the first-time stroke event; ^#^ethnicity group originated from East Malaysia (Iban, Bidayuh, Kadazan, etc.). Abbreviations: ACEIs = angiotensin-converting enzyme inhibitors; ARBs = angiotensin receptor blockers; ADM = antidiabetic medications; NIH=National Institute of health; OHA = oral hypoglycemic agent.

## Data Availability

The data related to the study have been provided in the article and are also available on request from the corresponding author.

## Supplementary Materials

Supplementary Materials The table includea those variables which were found insignificant in univariate or multivariate analysis.
