# Supplementary material for: Gender Differences and Risk Factors of Recurrent Stroke in Type 2 Diabetic Malaysian Population with History of Stroke: The Observation from Malaysian National Neurology Registry
Source: J Diabetes Res. 2019 Dec 11;2019:1794267. doi: 10.1155/2019/1794267 (PMC6927021; doi:10.1155/2019/1794267)
Supplement: Supplementary Materials — The table includea those variables which were found insignificant in univariate or multivariate analysis. [file 1794267.f1.docx]

|  | **Female** | | | **Male** | | | |
| --- | --- | --- | --- | --- | --- | --- | --- |
|  | **No**  **Recurrent** | **Had recurrent** | ***p***  **value** | **No recurrent** | **Had recurrent** | | ***p***  **value** |
| **Marital status** | | | | | | | |
| *Single* | 32(88.9) | 4(11.1) | 0.224 | 50(96.2) | | 2(3.8) | 0.458 |
| *Married* | 1885(95.1) | 97(4.9) |  | 1963(95.2) | | 98(4.8) |  |
| *Divorced* | 14(100) | 0(0.0) |  | 16(94.1) | | 1(5.1) |  |
| *Widowed* | 92(95.8) | 4(4.2) |  | 17(94.4) | | 1(5.6) |  |
| *Unknown* | 148(97.4) | 4(2.6) |  | 188(96.9) | | 6(3.1) |  |
| **Types of stroke (WHO classification)** | | | | | | | |
| *Ischemic stroke* | 1829(95.1) | 95(0.4) | 0.785 | 1870(95.3) | | 92(4.7) | 0.193 |
| *Intracerebral haemorrhage* | 255(96.2) | 10(3.8) |  | 285(95.0) | | 15(5.0) |  |
| *Subarachnoid haemorrhage* | 7(100) | 0(0.0) |  | 5(100.0) | | 0(0.0) |  |
| *Transient ischemic stroke* | 59(93.7) | 4(6.3) |  | 55(98.2) | | 1(1.8) |  |
| *Unclassified* | 20(100) | 0(0.0) |  | 19(100.0) | | 0(0.0) |  |
| **Known risk factors of first-time stroke** | | | | | | | |
| **Body mass index** |  |  |  |  | |  | 0.570 |
| *Normal weight* | 406(96.0) | 17(4.0) | 0.328 | 349(95.6) | | 16(4.4) |  |
| *Overweight* | 564(93.8) | 37(6.2) |  | 576(96.2) | | 23(3.8) |  |
| *Obese* | 1140(95.6) | 52(4.4) |  | 1228(95.0) | | 65(5.0) |  |
| *Underweight* | 49(94.2) | 3(5.8) |  | 54(93.1) | | 4(6.9) |  |
| **Atrial fibrillation** | 65(95.6) | 3(4.4) | 0.589 | 71(98.6) | | 1(1.4) | 0.257 |
| **No atrial fibrilation** | 2106(95.2) | 106(4.8) |  | 2163(95.3) | | 107(4.7) |  |
| **Alcohol intake** | 6(100.0) | 0(0.0) | 0.745 | 56(96.6) | | 2(3.4) | 0.494 |
| **No alcohol intake** | 2165(95.2) | 109(4.8) |  | 2178(95.4) | | 106(4.6) |  |
| **Hyperuricemia** | 76(92.7) | 6(7.3) | 0.195 | 99(98.0) | | 2(2.0) | 0.325 |
| **No Hyperuricemia** | 2095(95.3) | 103(4.7) |  | 2135(95.3) | | 106(4.7) |  |
| **Family History of Stroke** | 112(94.1) | 7(5.9) | 0.341 | 122(93.1) | | 9(6.9) | 0.197 |
| **No family history of stroke** | 2059(95.3) | 102(4.7) |  | 2112(95.5) | | 99(4.5) |  |
| **Medication received prior to the first-time stroke event** |  |  |  |  | |  |  |
| *Antiplatelet* | 543(94.6) | 31(5.4) | 0.242 | 544(94.4) | | 32(5.6) | 0.210 |
| *No antiplatelet* | 1628(95.4) | 78(4.6) |  | 1690(95.7) | | 76(4.3) |  |
| *Anticoagulant* | 33(91.7) | 3(8.3) | 0.246 | 41(97.6) | | 1(2.4) | 0.415 |
| *No anticoagulants* | 2138(95.3) | 106(4.7) |  | 2193(95.3) | | 107(4.7) |  |
| *Beta blocker* | 353(94.9) | 19(5.1) | 0.414 | 296(95.8) | | 13(4.2) | 0.426 |
| *No Beta Blockers* | 1818(95.3) | 90(4.7) |  | 1938(95.3) | | 95(4.7) |  |
| *Alpha Blocker* | 48(92.3) | 4(7.7) | 0.235 | 57(100.0) | | 0(0.0) | 0.110 |
| *No Alpha Blocker* | 2123(95.3) | 105(4.7) |  | 2177(95.3) | | 108(4.7) |  |
| *Diuretics* | 304(91.8) | 27(8.2) | 0.003 | 237(95.2) | | 12(4.8) | 0.482 |
| *No Diuretics* | 1867(95.8) | 82(4.2) |  | 1997(95.4) | | 96(4.6) |  |
| *Lipid lowering drugs* | 746(94.6) | 43(5.4) | 0.162 | 659(95.8) | | 29(4.2) | 0.319 |
| *No Lipid Lowering drugs* | 1425(95.6) | 66(4.4) |  | 1575(95.2) | | 79(4.8) |  |
| **Medication received upon discharge from hospitalisation secondary to the first-time stroke event** |  |  |  |  | |  |  |
| Received medication upon discharge | 1905(95.0) | 101(5.0) | 0.076 | 1945(95.3) | | 95(4.7) | 0.463 |
| No medication upon discharge | 266(97.1) | 8(2.9) |  | 289(95.7) | | 13(4.3) |  |
| Received Antiplatelet upon discharge | 1605(94.8) | 88(5.2) | 0.067 | 1588(95.3) | | 79(4.7) | 0.366 |
| No Antiplatelet upon discharge | 566(96.4) | 21(3.6) |  | 646(95.7) | | 29(4.3) |  |
| Received anti-coagulant upon discharge | 138(94.5) | 8(5.5) | 0.398 | 149(96.8) | | 5(3.2) | 0.273 |
| No anti-coagulant upon discharge | 2033(95.3) | 101(4.7) |  | 2085(95.3) | | 103(4.7) |  |
| Received ACEIs upon discharge | 696(95.0) | 37(5.0) | 0.376 | 719(95.7) | | 32(4.3) | 0.330 |
| No ACEIs upon discharge | 1475(95.3) | 72(4.7) |  | 1515(95.2) | | 76(4.8) |  |
| Received ARB upon discharge | 30(96.8) | 1(3.2) | 0.559 | 42(100.0) | | 0(0.0) | 0.261 |
| No ARB upon discharge | 2141(95.2) | 108(4.8) |  | 2192(95.3) | | 108(4.7) |  |
| Received CCB upon discharge | 529(94.6) | 30(5.4) | 0.260 | 509(96.2) | | 20(3.8) | 0.180 |
| No CCB upon discharge | 1642(95.4) | 79(4.6) |  | 1725(95.1) | | 88(4.9) |  |
| Received beta blocker upon discharge | 245(96.1) | 10(3.9) | 0.309 | 273(97.8) | | 6(2.2) | 0.033 |
| No beta blocker upon discharge | 1926(95.1) | 99(4.9) |  | 1961(95.1) | | 102(4.9) |  |
| Received alpha blocker upon discharge | 46(95.8) | 2(4.2) | 0.595 | 67(95.7) | | 3(4.3) | 0.595 |
| No alpha blocker upon discharge | 2125(95.2) | 107(4.8) |  | 2167(95.4) | | 105(4.6) |  |
| Received lipid lowering drugs upon discharge | 1732(95.0) | 91(5.0) | 0.208 | 1694(95.8) | | 75(4.2) | 0.132 |
| No lipid lowering drugs | 439(96.1) | 18(3.9) |  | 540(94.2) | | 33(5.8) |  |
| **Glasgow Coma Scale: Eye Opening** |  |  |  |  | |  |  |
| *No response* | 114(95.8) | 5(4.2) | 0.436 | 119(99.2) | | 1(0.8) | 0.072 |
| *To pain only* | 74(97.4) | 2(2.6) |  | 52(100.0) | | 0(0.0) |  |
| *To verbal stimuli, command, speech* | 133(95.7) | 6(4.3) |  | 146(94.8) | | 8(5.2) |  |
| *Spontaneous* | 1824(95.0) | 95(5.0) |  | 1881(95.0) | | 98(5.0) |  |
| **Glasgow Coma Scale: Verbal response** |  |  |  |  | |  |  |
| *No response* | 260(95.2) | 13(4.8) | 0.527 | 268(97.8) | | 6(2.2) | 0.114 |
| *Incomprehensible sound* | 176(96.7) | 6(3.3) |  | 188(97.4) | | 5(2.6) |  |
| *Inappropriate words* | 58(95.1) | 3(4.9) |  | 51(94.4) | | 3(5.6) |  |
| *Confused conversation, but able to answer questions* | 113(95.8) | 5(4.2) |  | 101(96.2) | | 4(3.8) |  |
| *Oriented* | 1529(95.0) | 81(5.0) |  | 1591(94.7) | | 89(5.3) |  |
| **Glasgow Coma Scale: Intubated** | 33(91.7) | 3(8.3) | 0.246 | 58(98.3) | | 1(1.7) | 0.234 |
| ***Not intubated*** | 2138(95.3) | 106(4.7) |  | 2176(95.3) | | 107(4.7) |  |
| **Handedness** |  |  |  |  | |  |  |
| *Right* | 1828(95.0) | 97(5.0) |  | 1815(95.1) | | 93(4.9) |  |
| *Left* | 87(92.6) | 7(7.4) | 0.370 | 125(94.0) | | 8(6.0) | 0.343 |
| *Ambidextrous* | 13(100) | 0(0.0) |  | 17(89.5) | | 2(10.5) |  |
| *Not available* | 25(100) | 0(0.0) |  | 33(100.0) | | 0(0.0) |  |
| **Clinical manifestations apparent at baseline** |  |  |  |  | |  |  |
| Hemiparesis | 1534(95.3) | 75(4.7) | 0.508 | 1605(95.4) | | 78(4.6) | 0.824 |
| No hemiparesis | 580(94.6) | 33(5.4) |  | 587(95.1) | | 30(4.9) |  |
| Tetraparesis | 33(100) | 0(0.0) | 0.406 | 23(100.0) | | 0(0.0) | 0.323 |
| No tetraparesis | 2050(95.0) | 108(5.0) |  | 2132(95.2) | | 108(4.8) |  |
| **NIH stroke scale at baseline** |  |  |  |  | |  |  |
| **Questions** |  |  |  |  | |  |  |
| *Answer both questions* | 1079(94.6) | 62(5.4) | 0.188 | 1153(95.2) | | 58(4.8) | 0.545 |
| *Answer to only one question* | 106(94.6) | 6(5.4) |  | 105(96.3) | | 4(3.7) |  |
| *Not available* | 348(96.9) | 11(3.9) |  | 331(96.5) | | 12(3.5) |  |
| **Commands** |  |  |  |  | |  |  |
| *Performs both tasks* | 1123(94.7) | 63(5.3) | 0.253 | 1217(95.2) | | 61(4.8) | 0.407 |
| *Performs only one task* | 173(94.5) | 10(5.5) |  | 156(97.5) | | 4(2.5) |  |
| *Performs neither tasks* | 239(97.2) | 7(2.8) |  | 210(95.9) | | 9(4.9) |  |
| **Gaze** |  |  |  |  | |  |  |
| *Normal* | 1227(94.8) | 67(5.2) | 0.633 | 1302(95.0) | | 68(5.0) | 0.086 |
| *Partial gaze palsy* | 221(95.7) | 10(4.3) |  | 192(97.0) | | 6(3.0) |  |
| *Forced deviation, or total gaze paresis* | 66(97.1) | 2(2.9) |  | 69(100.0) | | 0(0.0) |  |
| **Facial palsy** |  |  |  |  | |  |  |
| *Normal* | 603(93.5) | 42(6.5) | 0.067 | 619(95.2) | | 31(4.8) | 0.836 |
| *Minor facial paralysis* | 686(95.8) | 30(4.2) |  | 695(95.6) | | 32(4.4) |  |
| *Partial facial paralysis* | 221(97.4) | 6(2.6) |  | 249(96.5) | | 9(3.5) |  |
| *Complete facial paralysis* | 33(97.1) | 1(2.9) |  | 33(94.3) | | 2(5.7) |  |
| **Arm strength -left** |  |  |  |  | |  |  |
| *No drift* | 1199(94.5) | 70(5.5) | 0.394 | 1305(94.7) | | 73(5.3) | 0.243 |
| *Drift down before 10 seconds* | 290(95.7) | 13(4.3) |  | 325(95.9) | | 14(4.1) |  |
| *Some effort against gravity* | 238(95.6) | 11(4.4) |  | 204(96.2) | | 8(3.8) |  |
| *No effort against gravity* | 164(96.5) | 11(4.4) |  | 11(94.9) | | 6(5.1) |  |
| *No movement* | 280(96.9) | 9(3.1) |  | 289(97.6) | | 7(2.4) |  |
| **Arm strength-right** |  |  |  |  | |  |  |
| *No drift* | 1283(94.7) | 72(5.3) | 0.316 | 1400(94.9) | | 76(5.1) | 0.590 |
| *Drift down before 10 seconds* | 270(94.4) | 16(5.6) |  | 256(96.6) | | 9(3.4) |  |
| *Some effort against gravity* | 213(97.3) | 6(2.7) |  | 195(96.5) | | 7(3.5) |  |
| *No effort against gravity* | 142(95.9) | 6(4.1) |  | 127(96.2) | | 5(3.8) |  |
| *No movement* | 263(96.7) | 9(3.3) |  | 256(95.9) | | 11(4.1) |  |
| **Leg strength -left** |  |  |  |  | |  |  |
| *No drift* | 1131(94.5) | 66(5.5) | 0.428 | 1277(94.5) | | 74(5.5) | 0.126 |
| *Drift down before 10 seconds* | 294(95.8) | 13(4.2) |  | 297(97.4) | | 8(2.6) |  |
| *Some effort against gravity* | 297(96.1) | 12(3.9) |  | 260(95.6) | | 12(4.4) |  |
| *No effort against gravity* | 221(95.3) | 11(4.7) |  | 176(95.7) | | 8(4.3) |  |
| *No movement* | 228(97.0) | 7(3.0) |  | 224(97.4) | | 6(2.6) |  |
| **Leg strength -right** |  |  |  |  | |  |  |
| *No drift* | 1180(94.8) | 65(5.2) | 0.721 | 1338(94.8) | | 74(5.2) | 0.186 |
| *Drift down before 10 seconds* | 281(94.9) | 15(5.1) |  | 276(96.8) | | 9(3.2) |  |
| *Some effort against gravity* | 294(96.1) | 12(3.9) |  | 222(94.5) | | 13(5.5) |  |
| *No effort against gravity* | 189(95.5) | 9(4.5) |  | 184(96.3) | | 7(3.7) |  |
| *No movement* | 227(96.6) | 8(3.4) |  | 214(97.7) | | 5(2.3) |  |
| **Ataxia** |  |  |  |  | |  |  |
| *Absent* | 1948(95.0) | 103(5.0) | 0.264 | 1967(95.3) | | 96(4.7) | 0.489 |
| *Ataxia in only one limb* | 161(97.6) | 4(2.4) |  | 205(94.9) | | 11(5.1) |  |
| *Ataxia in two limbs* | 62(96.9) | 2(3.1) |  | 62(98.4) | | 1(1.6) |  |
| **Sensory** |  |  |  |  | |  |  |
| *Normal* | 1471(94.8) | 80(5.2) | 0.134 | 1497(96.0) | | 63(4.0) | 0.173 |
| *Mild to moderate sensory loss* | 595(95.5) | 28(4.5) |  | 634(94.2) | | 39(5.8) |  |
| *Severe to complete sensory loss* | 105(99.1) | 1(0.9) |  | 103(94.5) | | 6(5.5) |  |
| **Language** |  |  |  |  | |  |  |
| *No aphasia* | 1504(94.7) | 85(5.3) | 0.066 | 1501(95.1) | | 78(4.9) | 0.444 |
| *Mild to moderate aphasia* | 277(95.8) | 12(4.2) |  | 338(96.8) | | 11(3.2) |  |
| *Severe aphasia* | 152(99.3) | 1(0.7) |  | 152(94.4) | | 9(5.6) |  |
| *Mute or global aphasia* | 238(95.6) | 11(4.4) |  | 243(96.0) | | 10(4.0) |  |
| **Dysarthria** |  |  |  |  | |  |  |
| *Normal* | 1097(94.5) | 64(5.5) | 0.244 | 1053(94.7) | | 59(5.3) | 0.147 |
| *Mild to moderate dysarthria* | 699(95.9) | 30(4.1) |  | 805(96.5) | | 29(3.5) |  |
| *Severe dysarthria or anarthria* | 375(96.2) | 15(3.8) |  | 376(94.9) | | 20(5.1) |  |
| **Inattention** |  |  |  |  | |  |  |
| *No abnormality* | 1633(94.9) | 87(5.1) | 0.311 | 1689(95.2) | | 85(4.8) | 0.738 |
| *Mild inattention* | 324(95.3) | 16(4.7) |  | 324(96.1) | | 13(3.9) |  |
| *Severe inattention* | 2171(95.2) | 109(4.8) |  | 221(95.7) | | 10(4.3) |  |
